# Supplementary material for: Transcriptome Analysis of Roots from Wheat (Triticum aestivum L.) Varieties in Response to Drought Stress
Source: Int J Mol Sci. 2023 Apr 14;24(8):7245. doi: 10.3390/ijms24087245 (PMC10139362; doi:10.3390/ijms24087245)
Supplement: Supplementary file 1 [file ijms-24-07245-s001.zip › Supplementary-figure.pdf.pdf]

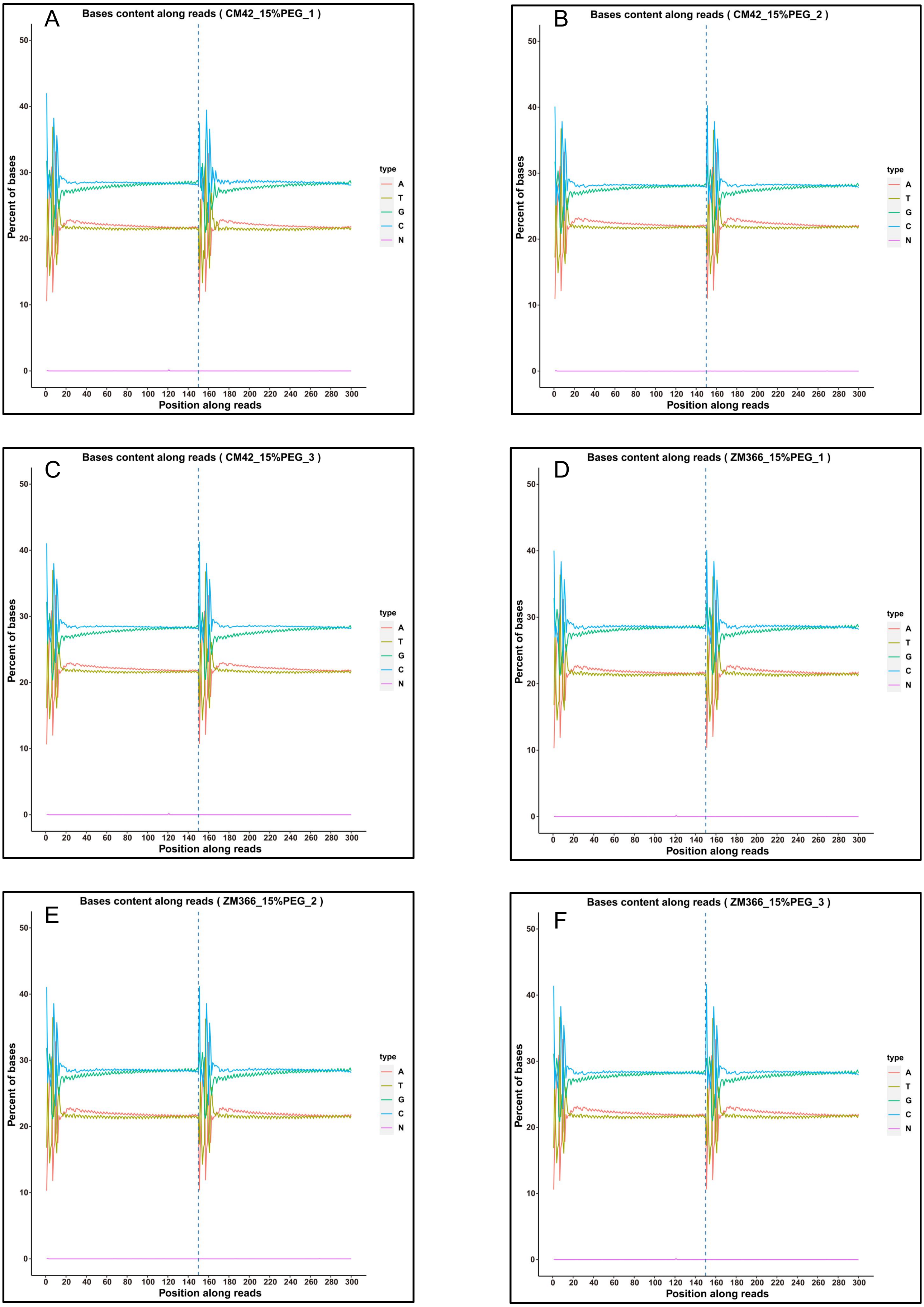

Figure S1. ZM366 and CM42 GC content statistics of sequencing results. In the figure, the x-coordinate is the base position of reads, and the y-coordinate is the percentage of the five base types of ATGCN. (A-C) CM42 is treated with 15% PEG-6000 three repeated experiments GC content statistics of sequencing results. (D-F) ZM366 is treated with 15% PEG-6000 three repeated experiments GC content statistics of sequencing results.

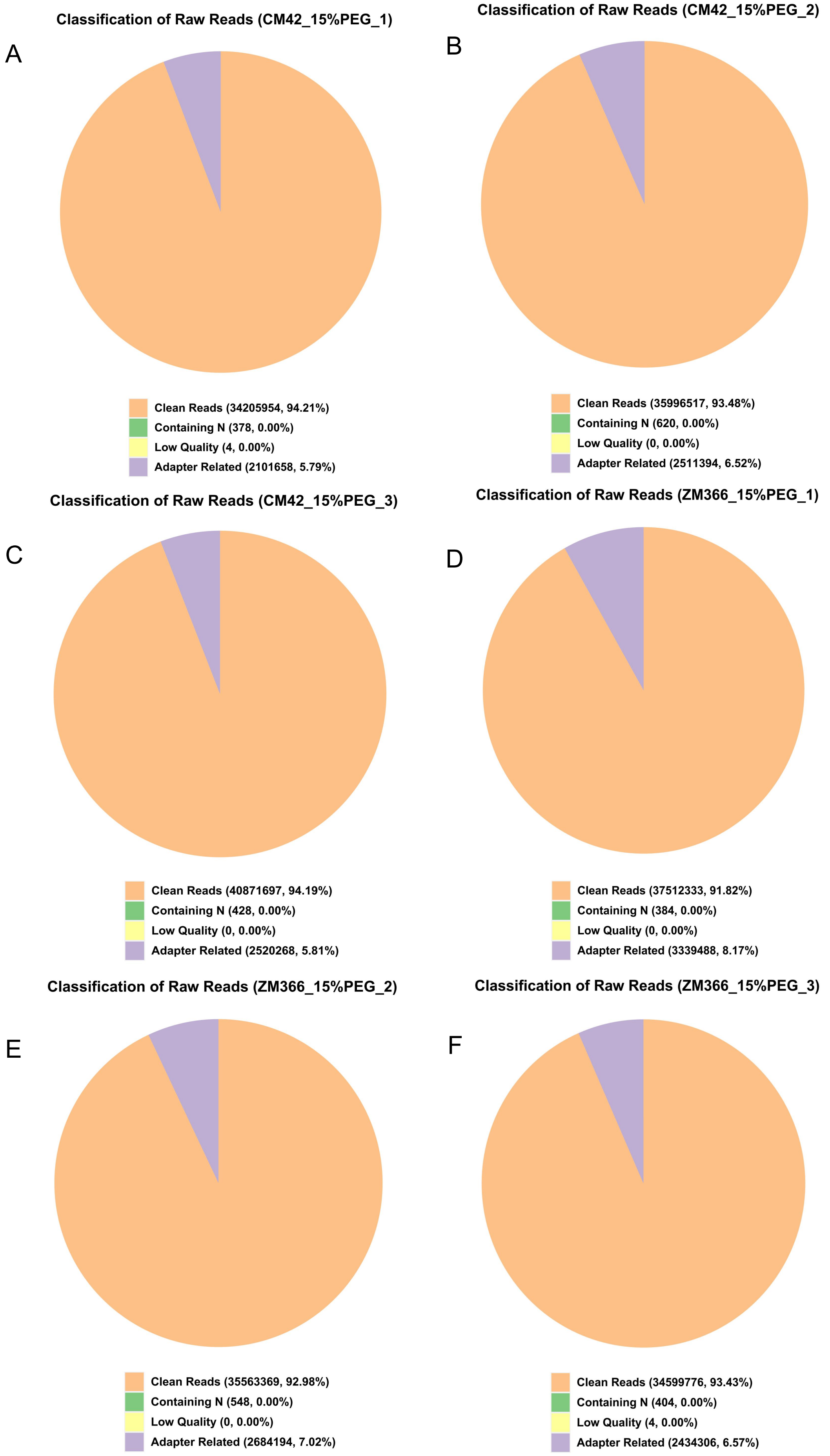

Figure S2. ZM366 and CM42 sequencing data filtering statistics**.** The different color ratios in the figure represent the proportions of different components respectively,including Clean reads,Low quality, Containing N, Adapter related (A-C) CM42 is treated with 15% PEG-6000 three repeated experiments sequencing data filtering statistics. (D-F) ZM366 is treated with 15% PEG-6000 three repeated experiments sequencing data filtering statistics.

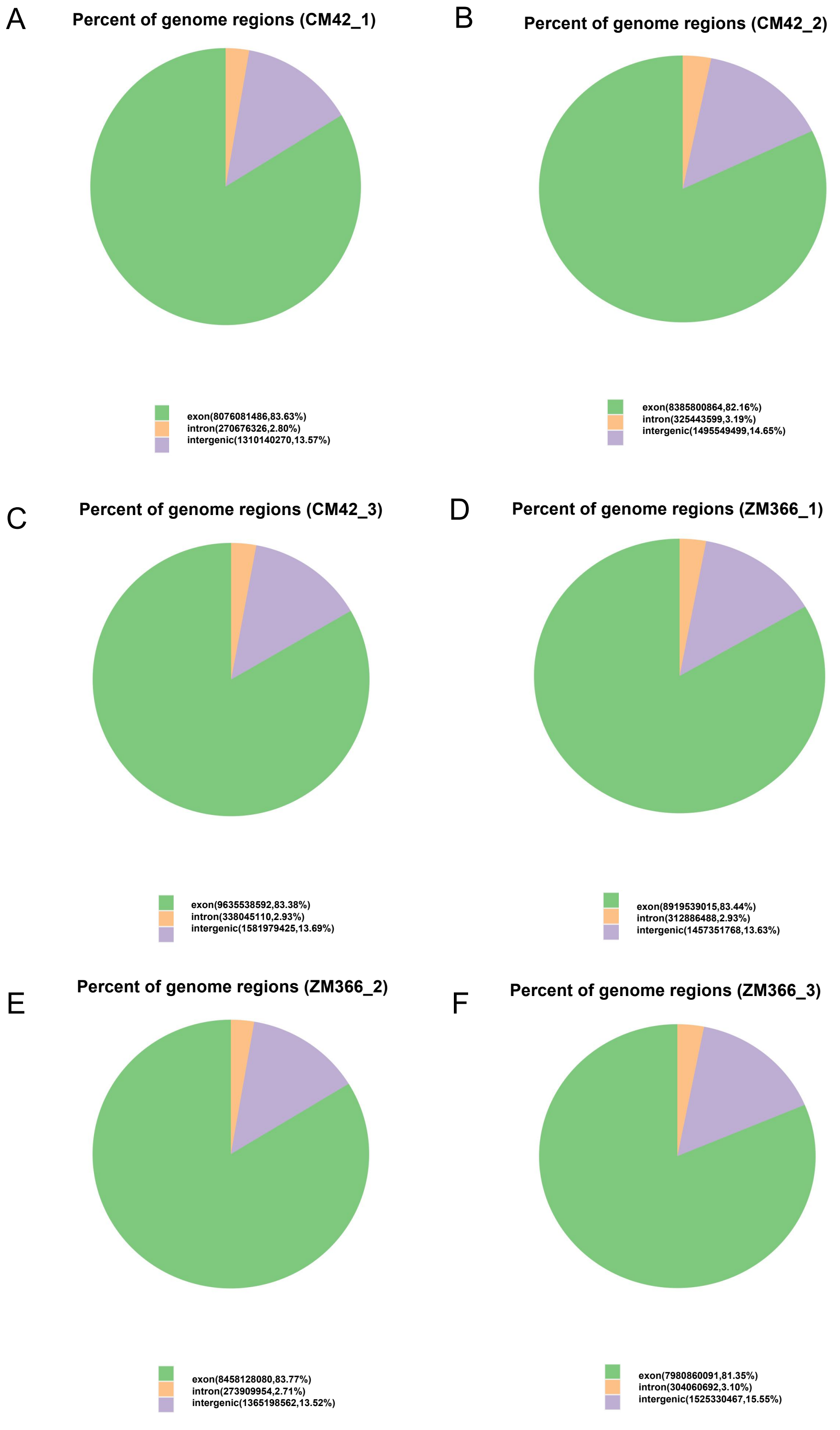

Figure S3. ZM366 and CM42 distribution of sequencing reads in genomic regions. Different color ratios in the figure respectively represent the ratio of reads to different regions, including exon, intron and intergentic. (A-C) CM42 is treated with 15% PEG-6000 three repeated experiments Distribution of sequencing reads in genomic regions. (D-F) ZM366 is treated with 15% PEG-6000 three repeated experiments Distribution of sequencing reads in genomic regions.

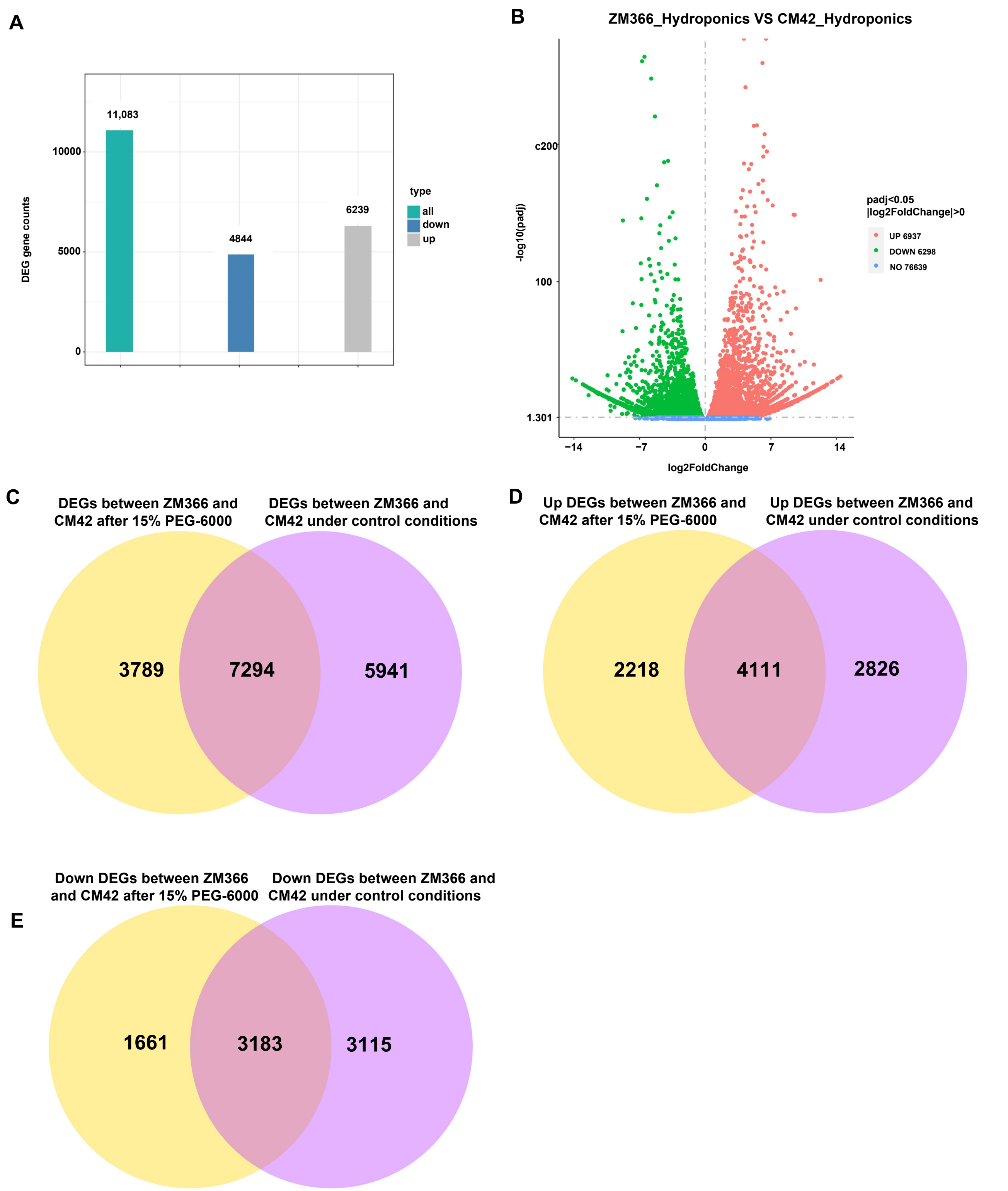

Figure S4. Differentially expressed genes in ZM366 and CM42 under control conditions and 15% PEG-6000. (A) Bar chart of ZM366 and CM42 differentially expressed genes. There were 11083 DEGs, of which 6239 genes were up-regulated and 4844 genes were down-regulated. (B) Volcano map of differentially expressed genes (DEGs) in ZM366 under control conditions-Hydroponics (ZM366_Hydroponics) and CM42 under control conditions-Hydroponics (CM366_Hydroponics). Up, 6937; down, 6298. (C) DEGs between ZM366 and CM42 under control conditions and 15% PEG-6000, 7294 DEGs were uniquely identified. (D) Up DEGs between ZM366 and CM42 under control conditions and 15% PEG-6000, 4111 DEGs were uniquely identified. (E) Down DEGs between ZM366 and CM42 under control conditions and 15% PEG-6000, 3183 DEGs were uniquely identified.
